# EXOME-WIDE STUDY IDENTIFIED 12 PLEIOTROPIC LOCI ASSOCIATED WITH ALZHEIMER’S AND CARDIOVASCULAR DISEASE RISKS

**DOI:** 10.1093/geroni/igac059.1728

**Published:** 2022-12-20

**Authors:** Yury Loika, Elena Loiko, Konstantin Arbeev, Eric Stallard, Anatoliy Yashin, Irina Culminskaya, Alexander Kulminski

**Affiliations:** Duke University, Durham, North Carolina, United States; Duke University, Durham, North Carolina, United States; Duke University, Durham, North Carolina, United States; Duke University, Durham, North Carolina, United States; Duke University, Durham, North Carolina, United States; Duke University, Durham, North Carolina, United States; Duke University, Durham, North Carolina, United States

## Abstract

Health of brain, heart and blood vessels are closely connected, which means that Alzheimer’s (AD) and cardiovascular (CVD) diseases may have overlapping etiologies including genetic component. Most of previous genetic association studies considered different traits (AD, CVD, and their risk factors) separately. The analysis of pleiotropic predisposition to these traits may shed light on the trait-specific mechanisms in protection against AD. We carried out pair-wise pleiotropic exome-wide association study (~250K common genetic variants) in predisposition to AD and each of 17 traits in a sample of 118K individuals from UK biobank. The analysis included seven qualitative traits (CVD, coronary-heart disease, type 2 diabetes, stroke, myocardial infarction, heart failure and hypertension), as well as their 10 risk factors (blood glucose, body-mass index, height, weight, 4 lipid traits, systolic and diastolic blood pressure). Fisher’s method and omnibus test were used in pleiotropic analyses. In addition to the APOE-TOMM40 locus, the analysis identified 12 genetic loci in which genetic polymorphisms demonstrated significant associations with AD at p≤5×10-4 and pleiotropic associations at genome-wide level, p≤5×10-8. The identified genes are involved in processes of phosphorylation; regulation of cell growth, differentiation, and brain development; signal transduction and neurotransmission; mitochondrial and cytoskeleton organization; regulation of gene expression, apoptotic processes, and adaptive immune response. Our results provide novel evidence supporting the hypothesis of complex pleiotropic mechanisms contributing to the development and progression of AD and provide more insights into understanding of underlying biological functions and regulatory mechanisms behind these effects.

